# Characteristics and Indications of Kawasaki Disease Among Infants Under 6 Months

**DOI:** 10.3389/fped.2020.00470

**Published:** 2020-08-14

**Authors:** Yunjia Tang, Xuan Li, Lei Cao, Ye Chen, Wenhua Yan, Qiuqin Xu, Haitao Lv

**Affiliations:** Department of Cardiology, Children's Hospital of Soochow University, Suzhou, China

**Keywords:** Kawasaki disease, characteristics, infants, indications, urinary tract infection, adenovirus infection

## Abstract

**Objectives:** To explore the characteristics of Kawasaki disease (KD) under 6 months and to investigate the possible indications of incomplete KD (iKD).

**Methods:** The medical records of KD patients hospitalized in Children's Hospital of Soochow University from January 2007 to December 2017 were retrospectively reviewed and analyzed. A total of 50 cases of urinary tract infection (UTI) and 50 cases of adenovirus (ADV)-infected patients under 6 months that were age and gender-matched with the main complaint of high fever were selected as controls.

**Results:** A total of 1,872 KD patients were enrolled. Among them, 194 (10.4%) were infantile patients under 6 months. There were 72 (37.1%) and 494 (29.4%) iKD in patients younger and older than 6 months, respectively (*P* < 0.05). Although patients under 6 months had a shorter fever duration before immunoglobulin (IVIG) treatment, a larger proportion of these patients had IVIG resistance and coronary artery lesions. They also tended to have higher platelet (PLT) counts and C-reactive protein (CRP) and lower hemoglobin (Hb), percentage of neutrophils (N%), albumin and serum sodium. When we compared iKD under 6 months with UTI and ADV-infected patients, significant differences were found in white blood cells (WBC), Hb, PLT, CRP, N% and serum albumin (*P* < 0.05). After adjusting the confounders, Hb < 105.5 g/L, CRP > 22.7 mg/L, N% > 47.4 and PLT > 496 × 10^9^/L were indications of iKD when compared with ADV infection (area under the curve [AUC]: 0.872, 0.939, 0.707, and 0.684, respectively). N% > 51.8 and albumin < 39.0 g/L were indications of iKD when compared with UTI (AUC: 0.627 and 0.832, respectively).

**Conclusions:** Infantile KD patients under 6 months had their own particularity. Laboratory variables could be good indications of iKD when compared with UTI and ADV infection.

## Introduction

Kawasaki disease (KD) is one of the most common immune vasculitis in children, which predominantly occurs in children under 5 years of age. Although more than 50 years have passed since its first report, the etiology of the disease still remains unclear. Epidemiological studies showed that ~20% of untreated KD developed coronary artery lesions (CAL) despite timely treatment, making the disease the most common cause of acquired heart diseases in developed countries (1). Thus, studies on risk factors of CAL in KD were amassed in recent years all around the world (2–5). Several studies had reached the consensus that younger age was a potential risk factor of CAL (4, 5).

Intravenous immunoglobulin (IVIG) together with aspirin was generally considered the gold regimen in KD treatment. However, a certain proportion of patients didn't respond to initial IVIG treatment and were defined as IVIG resistance. As previously reported, infantile patients under 6 months was a risk factor for IVIG resistance (6, 7) and thus, had a higher tendency to CAL development (5, 8). Moreover, it was also pointed out that despite timely treatment, infantile patients under 6 months were prone to being incomplete cases and developing CAL (9).

Actually, some of the infantile patients might only present with unexplained fever in the absence of other manifestations (1), making the diagnosis of the disease in a dilemma. On the other hand, it was sometimes quite difficult to differentiate KD from other infectious diseases, such as adenovirus (ADV) infection and urinary tract infection (UTI) due to clinical similarities of persistent high fever and conjunctivitis or pyuria.

Overall, it is of great importance and interest to investigate the characteristics of these young incomplete KD (iKD). However, limited studies were focused on infantile KD all around the world (9–15), let alone infantile iKD under 6 months in the east part of China. In the present study, we aimed to explore the characteristics of KD infants aged under 6 months in Suzhou, East China. Moreover, we tried to compare the differences in febrile patients diagnosed with UTI and ADV infection, and to seek a commonly used laboratory variable that was indicative of iKD.

## Methods

### Patients Enrolled

We retrospectively reviewed the medical records of patients hospitalized in the Department of Cardiology in Children's Hospital of Soochow University with a main diagnosis of KD during Jan 2007 and Dec 2017. Patients who received initial IVIG in other hospitals and who refused IVIG infusion were excluded. Besides, we retrospectively collected 50 cases of UTI and 50 cases of ADV-infected patients under 6 months with the main complaint of high fever as febrile controls. The study was approved by the Ethics Committee of Children's Hospital of Soochow University.

### Diagnosis

The diagnosis of KD was confirmed when a patient was febrile for ≥5 days, together with four of the following five characteristics: (1) Rash, (2) Bilateral conjunctive injection, (3) Cervical lymphadenopathy, (4) Changes of the extremities, (5) Oral mucosal changes. iKD was diagnosed according to the guideline when a patient had two or three compatible clinical characteristics (16). IVIG resistance was referred to when a patient had a persistent or recrudescent fever lasting for more than 36 h after the initiation of IVIG (16). ADV infection was confirmed when the nasal aspirate specimens of the patients were ADV positive by direct immunofluorescence assay using murine monoclonal antibodies (Chemicon). Patients with nasal aspirate specimens of bacterial infection were excluded. UTI was considered as more than 100,000 colony counts for one single pathogen or 10,000 colony counts in the symptomatic child with active urinalysis.

### Laboratory Data Collection

Data regarding epidemiologic, clinical, laboratory, and echocardiographic characteristics were documented. Laboratory data regarding white blood cell (WBC) count, percentage of neutrophils (N%), hemoglobin (Hb), platelet (PLT) count, C-reactive protein (CRP), serum albumin, serum sodium, alanine aminotransferase (ALT), and aspartate aminotransferase (AST) were obtained within 24 h on admission.

### Echocardiographic Findings

A routine two-dimensional echocardiographic evaluation was performed before IVIG infusion and was repeated within 2 weeks of disease onset. Echocardiographic findings were based on a written cardiologist's evaluation and cardiac ultrasound reports. CAL was defined when the internal lumen diameter was >3 mm in children <5 years old or >4 mm in children ≥5 years old (coronary artery dilations); if the internal diameter of a segment measured ≥1.5 times that of an adjacent segment (coronary artery dilations); or if the coronary lumen was clearly irregular. Giant coronary artery aneurysm (CAA) was defined when the internal lumen diameter was >8 mm. We chose the largest internal lumen dimension during the disease as the coronary status.

### Statistical Analysis

All statistical analyses were performed using SPSS 22.0 software (IBM, Armonk, NY, USA). Continuous variables with normal or skew distribution were presented as median with mean ± standard deviation (SD) or median with quartiles as appropriate. Categorical data were presented as numbers with percentages. Fisher's exact or χ^2^-test for categorical data was used to compare proportions between groups. For numerical comparisons between two groups, Student *t*-test and Wilcoxon Manny Whitney test were used in parametric and non-parametric tests as appropriate. For comparisons among three groups, ANOVA or Kruskal-Wallis *H*-test was used. Bonferroni *post-hoc* test was used to compare every two groups among the three groups. We chose laboratory variables with significant differences by Bonferroni *post-hoc* test into further multivariate logistic regression to analyze laboratory indications for iKD. Receiver operating characteristic (ROC) curves were used to evaluate the capacity of the indicative parameters. In order to compare CAL in patients younger and older than 6 months, IVIG resistance, iKD, gender and delayed treatment were included to adjust the potential confounders. Statistical significance was defined as a *P* < 0.05.

## Results

### Comparisons of Clinical Characteristics

**Figure 1:**
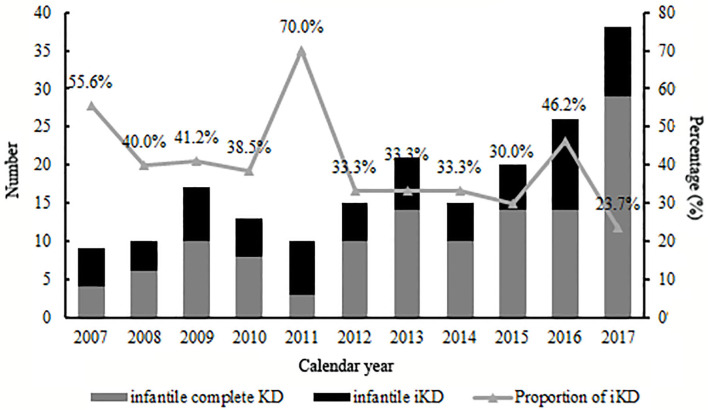
Number of infantile patients under 6 months with Kawasaki disease, by calendar year.

A total of 1,872 KD patients met the criteria and were enrolled in the present study. 566 (30.2%) were iKD and 1,306 (69.8%) were complete KD. Totally, 194 (10.4%) patients were under 6 months, among which 72 (37.1%) were iKD. As shown in Figure 1, total infantile KD and complete infantile KD were increasing stably during the period with some fluctuations, leading to a decreasing proportion of iKD with some variations out of expectations. The median ages of patients younger and older than 6 months were 4.0 months (mean ± SD [3.9 ± 1.0] months) and 21.0 months (mean ± SD [28.8 ± 22.1] months). Among KD under 6 months, the youngest was a 49-day male patient. There were 120 males and 74 females with a male to female ratio of 1.62:1. Sixteen patients received IVIG after the 10th day of fever with a patient being treated until the 18th day. The comparisons of clinical characteristics between patients younger and older than 6 months are shown in Table 1. Although it seemed that patients under 6 months were earlier to be treated with IVIG (6 [6.31 ± 2.26] vs. 6 [6.72 ± 2.38], *P* < 0.05), a higher proportion of patients had IVIG resistance (8.2% vs. 4.7%, *P* < 0.05). Patients under 6 months had lower incidences of cervical lymphadenopathy, oral mucosal changes, changes of extremities and perineal desquamation (*P* < 0.05). Regarding the laboratory variables, these young KD tended to have higher PLT counts and CRP and lower Hb, N%, albumin and serum sodium (*P* < 0.05). No death was noted during the period.

**Table 1:** Clinical and laboratory characteristics of patients in Kawasaki disease (KD).

**Characteristics**	**KD younger than 6 months (*n* = 194)**	**KD older than 6 months (*n* = 1,678)**	***P*-value**
Males, *n* (%)	120 (61.9)	1,070 (63.8)	0.601
iKD, *n* (%)	72 (37.1)	494 (29.4)	0.028
IVIG resistance, *n* (%)	16 (8.2)	79 (4.7)	0.033
Delayed IVIG treatment after the 10th day of illness, *n* (%)	16 (8.2)	150 (8.9)	0.748
Days before IVIG, median (mean ± SD)	6 (6.3 ± 2.3)	6 (6.7 ± 2.4)	0.022
Total fever duration, days, median (mean ± SD)			
Rash, *n* (%)	155 (79.9)	1,292 (77.0)	0.361
Conjunctival injection, *n* (%)	165 (85.1)	1,455 (86.7)	0.522
Cervical lymphadenopathy, *n* (%)	96 (49.5)	1,207 (71.9)	<0.001
Oral mucosal changes, *n* (%)	164 (84.5)	1,525 (90.9)	0.005
Changes of extremities, *n* (%)	83 (42.8)	1,266 (75.4)	<0.001
Perineal desquamation, *n* (%)	100 (51.5)	616 (36.7)	<0.001
Laboratory data, median (mean ± SD)			
White blood cells, × 10^9^/L	14.9 (11.1, 19.6)	14.2 (11.2, 18.0)	0.084
Hemoglobin, g/L	101.0 (100.5 ± 11.1)	109.0 (109.6 ± 13.7)	<0.001
Platelet, × 10^9^/L	420.0 (357.5, 512.5)	361.0 (284.0, 453.0)	<0.001
C-reactive protein, mg/L	74.6 (47.6, 117.7)	62.3 (33.0, 99.9)	0.001
Serum sodium, mmol/L	134.2 (134.2 ± 2.9)	135.0 (134.7 ± 3.0)	0.039
Percentage of neutrophils, %	60.3 (50.5, 71.5)	66.6 (55.4, 76.4)	<0.001
ALT, U/L	22.8 (15.4, 39.4)	23.2 (13.4, 62.6)	0.746
AST, U/L	32.9 (23.4, 51.5)	32.7 (25.1, 47.7)	0.816
Albumin, g/L	37.7 (37.5 ± 6.7)	39.2 (39.1 ± 8.6)	<0.001

*SD, standard deviation; ALT, alanine aminotransferase; AST, aspartate aminotransferase; IVIG, intravenous immunoglobulin*.

### Echocardiographic Findings

CAL occurred in 70 (36.1%) KD under 6 months and 473 (28.2%) KD older than 6 months, respectively (*P* = 0.022). In order to better describe CAL incidences of different ages, we further grouped patients who were older than 6 months into 6–12, 13–36, 37–60, and over 61 months. We found 133 (29.0%) aged 6–12 months, 237 (29.6%) aged 13–36 months, 60 (23.6%) aged 37–60 months and 43 (25.9%) aged over 60 months had CAL (*P* = 0.056).

Among the 70 patients with CAL under 6 months, 18 patients had either coronary artery dilation >3 mm, including left main artery involvements in 11 patients, right main artery involvements in 12 patients, and anterior descending artery involvements in 5 patients. 65 (33.5%) patients had an irregular coronary lumen. 23 (11.9%) patients had an internal diameter of a segment that measured ≥1.5 times that of an adjacent segment. Among the patients whose coronary artery dilation >3 mm, three patients had coronary artery dilation >6 mm with the largest coronary artery dimension to be 12 mm in the left main coronary artery in a 4-month old boy. CAL occurrence was significantly related to male (*P* = 0.039) and delayed IVIG treatment (*P* = 0.022), but not IVIG resistance (*P* = 0.228).

We further compared the coronary artery status among the patients younger and older than 6 months, and found significant differences in the incidences of CAL and irregular coronary lumen (*P* = 0.022 and 0.001, see Table 2). No significant differences were found regarding the incidences of coronary artery dilation, giant CAA, as well as the dimensions of coronary artery dilation. However, after adjusting the potential confounders, significant differences were found in the incidences of CAL, coronary artery dilation and irregular coronary lumen (*P* = 0.020 for CAL, 0.007 for coronary artery dilation, 0.409 for giant CAA, and 0.004 for irregular coronary lumen, respectively).

**Table 2:** Echocardiographic characteristics of patients in Kawasaki disease (KD).

**Echocardiographic Characteristics**	**KD younger than 6 months (*n* = 194)**	**KD older than 6 months (*n* = 1,678)**	***P*-value**
CAL, *n* (%)	70 (36.1)	473 (28.2)	0.022
Coronary artery dilation, *n* (%)	18 (9.3)	274 (16.3)	0.473
Irregular coronary lumen, *n* (%)	65 (33.5)	386 (23.0)	0.001
Giant CAA, *n* (%)	2 (1.0)	7 (0.4)	0.238
Dimensions of dilations, mm, median (mean ± SD)			
Left main artery	3.2 (4.3 ± 2.9)	3.2 (3.5 ± 0.6)	0.344
Right main artery	3.4 (3.8 ± 1.0)	3.4 (3.8 ± 1.0)	0.834
Left anterior descending artery	4.2 (4.8 ± 2.0)	3.6 (4.1 ± 1.4)	0.414

CAL, coronary artery lesions; CAA, coronary artery aneurysm.

### Comparisons Among KD, ADV-Infected and UTI Patients

We further compared the characteristics of iKD patients under 6 months with 50 cases of ADV-infected and 50 cases of UTI patients that were age and gender-matched. We found WBC, Hb, PLT, CRP, N%, and serum albumin were significantly different among the three groups. Hb and albumin were the lowest in KD patients. WBC and CRP were the lowest in ADV-infected patients while there was no significant difference between KD and UTI patients. N% was the highest in KD patients. With regards to PLT, ADV-infected patients were the lowest, with no significant difference between KD and UTI patients. The results are shown in Table 3.

**Table 3:** Comparisons among incomplete Kawasaki disease (KD), adenovirus (ADV) infection and urinary tract infection (UTI) patients younger than 6 months.

**Characteristics**	**iKD (*n* = 72)**	**ADV (*n* = 50)**	**UTI (*n* = 50)**	***P*-value**
Age, months, median (mean ± SD)	4.0 (3.9 ± 1.0)	4.0 (4.0 ± 1.0)	3.0 (3.5 ± 1.7)	0.114
Male, *n*, %	44 (61.1)	40 (80.0)	32 (64.0)	0.075
White blood cells, × 10^9^/L	14.8 (10.1, 20.0)	10.3 (7.7, 12.8)*	15.4 (11.0, 21.1)^#^	<0.001
Hemoglobin, g/L	98.5 (96.8 ± 10.7)	117.5 (115.8 ± 12.4)*	106.0 (106.0 ± 12.0)*^#^	<0.001
Platelet, × 10^9^/L	443.5 (363.5, 575.5)	372.0 (291.0, 453.0)*	403.5 (324.0, 477.5)	0.007
C-reactive protein, mg/L	70.0 (35.6, 106.7)	1.8 (0.1, 12.8)*	58.5 (22.8, 96.7)^#^	<0.001
Percentage of neutrophils	56.4 (47.6, 67.2)	41.7 (21.7, 55.8)*	50.9 (32.3, 59.6)*	<0.001
ALT, U/L	21.8 (15.4, 39.4)	23.1 (15.2, 37.9)	23.8 (18.9, 30.3)	0.803
AST, U/L	32.3 (23.4, 51.5)	36.8 (18.7, 45.6)	34.0 (26.4, 46.2)	0.360
Albumin, g/L	37.6 (37.0 ± 3.2)	40.0 (40.2 ± 2.2)*	40.7 (41.0 ± 2.6)*^#^	<0.001

* *Significantly different when compared with KD*.

# *Significantly different when compared with ADV infection*.

*SD, standard deviation; ALT, alanine aminotransferase; AST, aspartate aminotransferase*.

### Indications of iKD When Compared With UTI and ADV Infection

In order to better elucidate the differences between iKD and the other two febrile diseases, we further tried to seek the indications of iKD when compared with UTI and ADV infection, respectively, in patients under 6 months. After adjusting the potential confounders, Hb, CRP, N%, and PLT were indications of iKD when compared with ADV infection (area under the curve [AUC]: 0.872, 0.939, 0.707, and 0.684, respectively) (Table 4). CRP was the best indication of iKD with a sensitivity of 0.855 and a specificity of 0.875, followed by Hb with a sensitivity of 0.819 and a specificity of 0.771. The AUC was 0.971 after combining all the indications with a sensitivity of 0.884 and a specificity of 0.956. On the other hand, N% and albumin were indications of iKD when compared with UTI (Table 5). Albumin had a better performance than N% (AUC:0.832 vs. 0.627). The AUC was 0.856 after combining N% and albumin with a sensitivity of 0.750 and a specificity of 0.840.

**Table 4:** Indicative value of the laboratory variables for differentiating incomplete Kawasaki disease (KD) from adenovirus (ADV) infection.

	**AUC**	**95% CI**	***P*-value**	**Cutoff points**	**Sensitivity**	**Specificity**
Hemoglobin	0.872	0.809–0.935	<0.001	105.5 g/L	0.819	0.771
C-reactive protein	0.939	0.901–0.978	<0.001	22.7 mg/L	0.855	0.875
Percentage of neutrophils	0.707	0.612–0.803	<0.001	47.4	0.764	0.600
Platelet	0.684	0.590–0.779	0.001	496 × 10^9^/L	0.403	0.936
Combination	0.971	0.947–0.996	<0.001	–	0.884	0.956

AUC, area under the curve; CI, confidence interval.

**Table 5:** Indicative value of the laboratory variables for differentiating incomplete Kawasaki disease (KD) from urinary tract infection (UTI).

	**AUC**	**95% CI**	***P*-value**	**Cutoff points**	**Sensitivity**	**Specificity**
Percentage of neutrophils	0.627	0.527–0.727	0.017	51.8	0.681	0.560
Albumin	0.832	0.761–0.902	<0.001	39.0 g/L	0.736	0.800
Combination	0.856	0.791–0.921	<0.001	–	0.750	0.840

*AUC, area under the curve; CI, confidence interval*.

## Discussion

It was of great importance to carry out an investigation in infantile KD due to the different characteristics of these patients. In this retrospective study, we found that although patients under 6 months had a shorter fever duration before IVIG treatment, a larger proportion of these patients had IVIG resistance and CALs. They also tended to have higher PLT and CRP, lower Hb, N%, albumin, and serum sodium. What was more, when infantile iKD were compared with ADV infection, Hb, CRP, N%, and PLT could work as indications of iKD. Otherwise, when we compared infantile iKD with UTI, N%, and albumin could work as indications of iKD.

In the present study, a total of 194 patients were under 6 months, and they accounted for 10.4% (194/1,872) of all KD patients. The result was similar to previous studies. Mastrangelo reported that 16.8% of patients were under 6 months in Italy (14) while Chang reported the proportion was 17% in Taiwan (15). Singh found the proportion was only 3.6% in India (12). All of them had a relatively small number of study patients. However, our study partly revealed the demographic characteristics in this area.

We had previously described an increasing number of KD patients in this area (17), with no focus on infantile KD. Here when we divided all 194 infantile KD into small groups according to calendar years, we found that although there was a stable increase of all infantile KD, the proportion of iKD seemed to be decreasing, which was inconsistent with the overall trend of KD as we reported (17). As the etiology and mechanisms of the disease were not fully understood, the divergences might be related to the pathogenesis in infants whose immune system was not well-developed. The high proportion in 2011 of 70% might be attributed to a relatively small number of iKD in this year. Thus, a multi-center study is warranted in the future. Otherwise, we speculated if the environmental factors or other infectious agents might play a role. However, we are unable to identify the facts because of unclear causes of the disease.

As reported before (13–15, 18, 19), we also found that a higher proportion of younger KD patients were iKD (37.1%), which was attributed to lower incidences of cervical lymphadenopathy, oral mucosal changes and changes of extremities in our study. Despite a higher incidence of iKD, fever duration before initial IVIG treatment was significantly shorter in younger infants (6.31 ± 2.26 vs. 6.72 ± 2.38, *P* = 0.022), indicating a better understanding and recognition of KD in younger infants. However, in another study which was carried out in South China (19), the authors found a longer fever duration before diagnosis in infantile KD under 3 months due to their incomplete presentations. We attributed this to the following reasons. Firstly, patients under 3 months had fewer clinical presentations than those in our study. Moreover, our study was more recent and physicians were more aware of iKD in recent years. Otherwise, it was reported that younger infants had a shorter fever duration before hospital admission because of more attention to them (13). Thus, IVIG was earlier to be used as soon as KD was diagnosed according to the latest guideline (1). However, in spite of the timely treatments in these young infants, a higher proportion of patients suffered from CALs and IVIG resistance, which was in line with the previous study (9). In that case, some more aggressive treatment regimens might be considered in these infantile KD.

Despite the lack of specific diagnostic laboratory variables, KD was considered as vascular inflammation and thus had higher inflammatory markers. Regarding the differences between patients younger and older than 6 months, we found PLT and CRP were higher, Hb, N%, albumin and serum sodium were lower in patients under 6 months. Our results were almost the same as Satoh's (13), although he set the section at 3 months. The results that younger patients had lower Hb and N% were partly caused by physiologic anemia in patients aged 2–3 months and a predominance of lymphocytes in childhood. It had been revealed that PLT, CRP, N%, serum sodium, and albumin were all risk factors of IVIG resistance in KD (7, 20, 21), which were possibly indicative of more severe inflammation. Although the specific mechanism of CAL was not well-elucidated and the relationship between ages and the laboratory variables was complicated (18), the incidence of CAL in younger patients was higher with no significant difference in giant CAA and dimension of the dilated coronary artery. Our results showed a lower incidence of coronary artery dilation that was different from that reported by other studies (9, 13), the divergences could be explained by coronary artery dilation definition larger than 3 mm when patients were younger than 3 years. In this sense, body surface adjusted-*z* score might be more appropriate.

ADV infection and UTI were considered as the differential diagnoses of KD and sometimes it was quite tough to distinguish them (22, 23), especially when the presentations of KD were absent in infantile patients. In recent years, a lot of biomarkers such as microRNAs were found to have important diagnostic value in KD (24). However, they were not available in most hospitals and the results were often delayed. We here compared several common laboratory variables in iKD, ADV, and UTI patients in order to better recognize the disease as soon as possible. We found Hb, CRP, N%, and PLT were indications when compared with ADV infection, among which CRP had the best performance. In this sense, when an infant with high fever and incomplete presentations such as conjunctival injection was admitted, indications of Hb < 105.5 g/L, CRP > 22.7 mg/L, N% > 47.4, and PLT > 496 × 10^9^/L would help the diagnosis of iKD. When we compared iKD with UTI, N% and albumin were indications. Similarly, when an infant with high fever, high levels of WBC and CRP was admitted, apart from the urine routine test, N% > 51.8 and albumin < 39.0 g/L would help the diagnosis of iKD.

Overall, infantile KD patients had their own particularity. Laboratory variables could be good indications of iKD when compared with UTI and ADV infection. Thus, echocardiography should be carried out in these infantile patients with persistent high fever and above indications, so that early IVIG treatment could be initiated. Moreover, additional anti-inflammatory treatments should target these patients and future studies will focus on the treatment regimens in KD infants under 6 months.

The present study has several limitations. Firstly, we used Japanese criteria for CAL rather than z score in the present study, which could lead to an underestimation of the CAL incidence in patients under 6 months. However, we were unable to calculate z scores because the height of most patients during the study period was not documented then. Secondly, due to the fact that ADV infection was more commonly seen in school-aged children (25), we chose a small number of ADV-infected and UTI patients as febrile controls in the present study. Thirdly, KD patients in the acute stage were reported to have elevated cardiac biomarkers such as troponin and pro-B-type natriuretic peptide (26). Unfortunately, we were unable to analyze troponin levels because it was not routinely tested and several different assays with different ranges had been applied during the 11-year period, and pro-B-type natriuretic peptide test hadn't been carried out in our hospital so far. Lastly, selection bias might exist because of the retrospective nature of the study. However, our study had a relatively large number of KD and iKD patients during the 11-year period and the results were robust.

## Data Availability Statement

The raw data supporting the conclusions of this article will be made available by the authors, without undue reservation.

## Ethics Statement

This study was approved by the Ethics Committee of Children's Hospital of Soochow University.

## Author Contributions

YT and XL were major contributors in writing the manuscript. LC analyzed and interpreted the patient data. WY and YC collected the data. QX and HL designed the study and reviewed the manuscript. All authors read and approved the final manuscript.

## Conflict of Interest

The authors declare that the research was conducted in the absence of any commercial or financial relationships that could be construed as a potential conflict of interest.

## Abbreviations

KD: Kawasaki disease
UTI: urinary tract infection
ADV: adenovirus
iKD: incomplete KD
IVIG: intravenous immunoglobulin
PLT: platelet counts
CRP: C-reactive protein
Hb: hemoglobin
N%: percentage of neutrophils
WBC: white blood cells
AUC: area under the curve
CAL: coronary artery lesions
CAA: coronary artery aneurysm
ALT: alanine aminotransferase
AST: aspartate aminotransferase.
